# Lessons learnt from a discontinued randomised controlled trial: adalimumab injection compared with placebo for patients receiving physiotherapy treatment for sciatica (Subcutaneous Injection of Adalimumab Trial compared with Control: SCIATiC)

**DOI:** 10.1186/s13063-018-2801-6

**Published:** 2018-07-31

**Authors:** Nefyn H. Williams, Alison Jenkins, Nia Goulden, Zoe Hoare, Dyfrig A. Hughes, Eifiona Wood, Nadine E. Foster, David Walsh, Dawn Carnes, Valerie Sparkes, Elaine M. Hay, John Isaacs, Kika Konstantinou, Dylan Morrissey, Jaro Karppinen, Stephane Genevay, Clare Wilkinson

**Affiliations:** 1 Department of Health Services Research, University of Liverpool, Waterhouse Block B, 1-5 Brownlow Street, Liverpool, L69 3GL UK; 2 School of Healthcare Sciences, Bangor University, Bangor, UK; 3 Arthritis Research UK Primary Care Centre, Research Institute for Primary Care and Health Sciences, Keele University, Keele, UK; 4 Arthritis Research UK Pain Centre and National Institute for Health Research Nottingham Biomedical Research Centre, School of Medicine, University of Nottingham, Nottingham, UK; 5 Centre for Primary Care and Public Health, Barts and the London School of Medicine and Dentistry, London, UK; 6 School of Healthcare Science, Cardiff University, Cardiff, UK; 7 National Institute for Health Research Newcastle Biomedical Research Centre, Newcastle upon Tyne Hospitals NHS Foundation Trust and Newcastle University, Newcastle upon Tyne, UK; 8 Centre for Sports and Exercise Medicine, William Harvey Research Institute, Barts and the London School of Medicine and Dentistry, Queen Mary University of London, London, UK; 9 Medical Research Centre Oulu, University of Oulu and Oulu University Hospital, Oulu, Finland; 10 University Hospitals of Geneva, Geneva, Switzerland

**Keywords:** Feasibility, Randomised controlled trial, Economic evaluation, Sciatica, Adalimumab, Anti-TNF-α, Biological agents

## Abstract

**Background:**

Adalimumab, a biological treatment targeting tumour necrosis factor α, might be useful in sciatica. This paper describes the challenges faced when developing a new treatment pathway for a randomised controlled trial of adalimumab for people with sciatica, as well as the reasons why the trial discussed was stopped early.

**Methods:**

A pragmatic, parallel group, randomised controlled trial with blinded (masked) participants, clinicians, outcome assessment and statistical analysis was conducted in six UK sites. Participants were identified and recruited from general practices, musculoskeletal services and outpatient physiotherapy clinics. They were adults with persistent symptoms of sciatica of 1 to 6 months’ duration with moderate to high level of disability. Eligibility was assessed by research physiotherapists according to clinical criteria, and participants were randomised to receive two doses of adalimumab (80 mg then 40 mg 2 weeks later) or saline placebo subcutaneous injections in the posterior lateral thigh. Both groups were referred for a course of physiotherapy. Outcomes were measured at baseline, 6-week, 6-month and 12-month follow-up. The main outcome measure was disability measured using the Oswestry Disability Index. The planned sample size was 332, with the first 50 in an internal pilot phase.

**Results:**

The internal pilot phase was discontinued after 10 months from opening owing to low recruitment (two of the six sites active, eight participants recruited). There were several challenges: contractual delays; one site did not complete contract negotiations, and two sites signed contracts shortly before trial closure; site withdrawal owing to patient safety concerns; difficulties obtaining excess treatment costs; and in the two sites that did recruit, recruitment was slower than planned because of operational issues and low uptake by potential participants.

**Conclusions:**

Improved patient care requires robust clinical research within contexts in which treatments can realistically be provided. Step changes in treatment, such as the introduction of biologic treatments for severe sciatica, raise complex issues that can delay trial initiation and retard recruitment. Additional preparatory work might be required before testing novel treatments. A randomised controlled trial of tumour necrosis factor-α blockade is still needed to determine its cost-effectiveness in severe sciatica.

**Trial registration:**

Current Controlled Trials, ISRCTN14569274. Registered on 15 December 2014.

## Background

Sciatica is a well-localised leg pain, attributed to nerve root irritation, that approximates the dermatomal distribution of the sciatic nerve down the posterior lateral aspect of the leg [1]. It is a common cause of pain and disability [2]. Although most cases resolve, up to 30% might have persistent troublesome symptoms after 1 year [3, 4]. Many patients whose symptoms settle relapse later [5]. Typical care pathways in the National Health Service (NHS) involve analgesia prescribed by a general practitioner (GP), referral for physiotherapy [6, 7], followed by more invasive treatment, such as epidural corticosteroid injection or disc surgery if symptoms persist [3, 4, 8]. However, the evidence for most of the non-surgical treatments is weak [9]; new treatment strategies are needed.

Sciatica caused by lumbar nerve root pain usually arises from a prolapsed intervertebral disc [3], which can compress the nerve root [10] but also releases pro-inflammatory factors such as tumour necrosis factor-α (TNF-α) that may lead to nerve sensitisation [11, 12]. Biological agents such as the monoclonal antibody adalimumab bind specifically to TNF-α receptors and might have beneficial effects on the inflamed nerve root in sciatica [13]. Two separate network meta-analyses of different treatment strategies for sciatica found that biological agents had the highest probability of having the best outcomes for pain, but with wide confidence intervals [14, 15]. A meta-analysis of biological agents for sciatica found insufficient evidence to change practice but sufficient evidence to suggest that clinically important benefit was possible and that a definitive randomised controlled trial (RCT) was warranted [16].

Sciatica is costly to society [17], and although biological agents are expensive, they may be cost-effective if they reduce the need for more expensive treatments such as disc surgery. Also, as patents expire, cheaper biosimilar drugs are becoming available [18]. Adalimumab is a TNF-α-blocking antibody that is administered by subcutaneous injection, with two doses given 2 weeks apart, and should inhibit TNF-α for at least 4 weeks. Adalimumab dosing for psoriasis or Crohn’s disease uses 80-mg followed by 40-mg subcutaneous injections [19]; the same dosing strategy is proposed in sciatica.

### Objectives

The aims of the RCT were to evaluate the effectiveness and cost-effectiveness of injections of adalimumab plus physiotherapy compared with placebo injection of 0.9% sodium chloride plus physiotherapy for patients with sciatica for whom first-line primary care treatment had failed. However, the RCT was discontinued because of lack of progress. The aim of this paper is to explore the reasons for this and make recommendations to inform other researchers.

## Methods

This was designed as a pragmatic, multi-centre RCT with blinded (masked) participants, clinicians, outcome assessment and statistical analysis, with concurrent economic evaluation and internal pilot. The Wales Research Ethics Committee (REC) 3 granted approval on 27 May 2015 (15/WA/105), and clinical trial authorisation from the Medicines and Healthcare products Regulatory Agency (MHRA) was granted on 15 April 2015 (21996/0002/001-0001). The setting was the NHS in England and Wales, with five collaborating university centres (designated 1–5). We aimed to recruit from six NHS sites overseen by these five centres (designated A–F). Each collaborating centre would oversee a number of patient identification centres, which consisted of general medical practices, local musculoskeletal services and outpatient physiotherapy clinics. Patients were identified in three ways:

1. By their GP
2. Following a search of the general practice patient record database
3. After referral to local musculoskeletal services

**Fig. 1:**
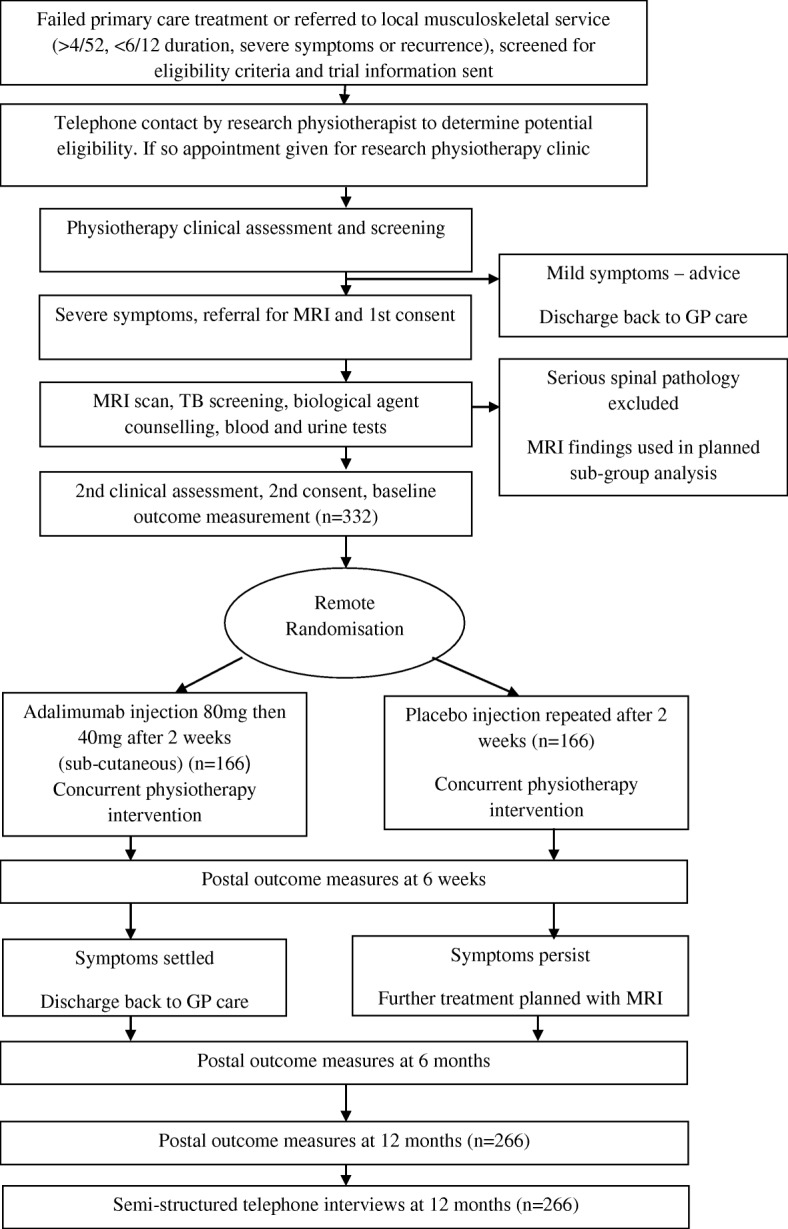
Trial flowchart. *GP* General practitioner, *MRI* Magnetic resonance imaging, *TB* Tuberculosis

Patients were invited to participate by letter. Those who were interested were contacted by telephone for pre-screening, and if they fitted the inclusion criteria, were given an appointment in a research clinic run by a research physiotherapist. At this research clinic, all potential participants were screened by the research physiotherapist for eligibility. If eligible, participants had blood tests, tuberculosis (TB) screening, biological agents counselling, and magnetic resonance imaging (MRI) to exclude serious spinal pathology. If they were still eligible, at a second screening assessment 2–3 weeks later, informed consent was obtained for trial entry and randomisation (Fig. 1).

### Inclusion criteria

- Clinical features of sciatica
  - ○ Leg pain worse than or as bad as back pain
  - ○ Unilateral leg pain approximating a dermatomal distribution
  - ○ Positive neural tension test such as straight leg raise test restricted < 50 degrees by leg pain, or positive femoral stretch test, or muscle weakness, or loss of tendon reflex, or loss of sensation in a dermatomal distribution
- 18 years of age or older
- Persistent symptoms for ≥ 4 weeks and < 6 months
- Moderate to high severity (≥ 30) on Oswestry Disability Index (ODI)

### Exclusion criteria

- Unable to undergo MRI
- Serious pathology
- Neurological deficit requiring urgent spinal surgery assessment
- Contralateral leg pain extending below the inferior gluteal margin
- Widespread pain throughout the body
- Prior use of biological agents within previous 6 months
- Previous lumbar spinal surgery
- Contraindications to adalimumab injection
- Unable to give informed consent

### Randomisation

Secure web-based randomisation was performed using a dynamic adaptive randomisation algorithm [20] to protect against subversion while ensuring that the trial maintained good balance to the allocation ratio of 1:1, both within each stratification variable and across the trial. Participants were stratified by (1) treatment centre and (2) presence of neurological signs (motor weakness or sensory loss).

### Interventions

All participants were randomised to receive an 80-mg adalimumab subcutaneous injection followed 2 weeks later by a 40-mg injection in the posterior thigh, or an equivalent volume of 0.9% sodium chloride. Both groups received a concurrent course of physiotherapy over a period of 12 weeks [21, 22].

### Internal pilot trial phase

The internal pilot trial phase aimed to assess the feasibility of the trial procedures, recruitment and retention rates, based on the first 50 participants recruited. The stopping criteria at the end of this internal pilot were recruitment which failed to reach 80% of the planned recruitment rate target, dropouts exceeding 20%, or more than one centre failing to commence recruitment.

### Outcome measures

The baseline questionnaire was administered by research physiotherapists. We planned to send postal questionnaires at 6 weeks, 6 months and 12 months post-randomisation. The primary clinical outcome was back pain-related disability using the ODI [23] measured at 12 months, which has evidence of validity for sciatica as well as back pain. The primary economic outcome was quality-adjusted life-year calculated from the EuroQol 5-level EQ-5D version (EQ-5D-5L) [24].

#### Condition-specific outcomes

- Oswestry Disability Index [23]
- Leg pain version of the Roland-Morris Disability Questionnaire [25, 26]
- Sciatica Bothersomeness Index [27]
- Pain location using a pain manikin [28]

#### Generic outcomes

- EuroQol EQ-5D-5L [24]
- Global assessment of change since baseline

#### Psychological outcome

- Hospital Anxiety and Depression Scale [29]

#### Use of health care and social care services

- Resource use questionnaire [30, 31]

#### Process measures (potential predictors and mediators of outcome)

- Keele STarT Back Risk Screening Tool [32]
- Pain trajectory (based on a single question) [33]
- Pain Self-Efficacy Questionnaire [34]
- Tampa Scale for Kinesiophobia [35]

### Sample size

In order to detect an effect size of 0.4 with 90% power, 5% significance and 80% retention rate, 332 patients would have needed to be recruited.

### Written qualitative comments

After the trial funding was withdrawn because of slow progress, the trial management team and all sites were asked to reflect on what worked and what did not work within the trial. Written comments were collated by the trial manager (AJ) and the chief investigator (NHW) and grouped into themes.

## Results

### Trial progress

**Fig. 2:**
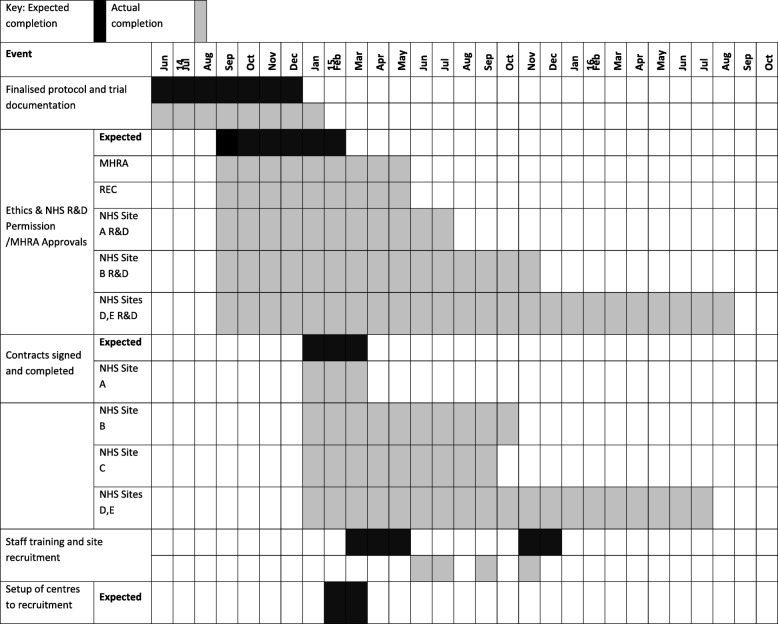
Trial timetable. *MHRA* Medicine and Healthcare products Regulatory Agency, *NHS* National Health Service, *R&D* Research and Development, *REC* Research ethics committee

Trial progress is compared with what was planned, as shown in Fig. 2. The letter of notification of funding was received on 11th August 2014. The trial documentation for the regulatory approval was in place in December 2014. Regulatory approval was obtained from the MHRA on 15th April 2015 and from the REC on 27th May 2015. There were long delays in signing contracts with University 4 and NHS sites D and E, and contracts were never signed with University 5 and NHS site F (Table 1). There were delays in obtaining the excess treatment costs (ETCs) for some sites in England. University 3 and NHS site C withdrew from the trial in February 2016. The trial initially opened to recruitment on 8th December 2015 at NHS sites A and B, with NHS sites D and E opening to recruitment on 11th August 2016. The trial was closed early on 23rd September 2016 because of poor recruitment.

**Table 1:** Time taken to sign subcontracts

University or NHS site	Date subcontract signed	Time to sign in days
NHS Site A	8th April 2015	9
University 2	7th July 2016	378
NHS Site B	20th October 2015	197
University 3	24th July 2015	115
NHS Site C	14th August 2015	131
University 4	2nd June 2016	427
NHS Site D	7th July 2016	459
NHS Site E	7th July 2016	459
University 5	Never signed	> 550
NHS Site F	Never signed	> 550

*NHS* National Health Service

### Contracting

The main contract was between the funder (NIHR Health Technology Assessment) and University 1, which was one of the centres and also acted as sponsor. Subcontracts between University 1 and the participating centres and sites concerning roles and responsibilities and the available funding were a major issue. Initial subcontract templates were drafted in November 2014 but could not proceed further until the main contract and finances were agreed with the funder in February 2015. Draft subcontracts were sent to the relevant parties from University 1 contracts department on 31st March 2015. The time taken to sign contracts is shown in Table 1.

There were protracted discussions about the nature and content of the subcontracts, as well as the division of responsibilities between the academic partners and the NHS sites. In the final adopted model, the subcontracts were between the NHS sites and the sponsor, with universities providing academic support rather than taking on contractual responsibilities [36].

There were initial discussions about whether to have a tripartite subcontract between University 1, University 4 and NHS site D, but after further discussion it was agreed that University 1 would have separate subcontracts. In addition, physiotherapy services were provided by two NHS sites, and separate subcontracts were needed for each. Two clinical rheumatology services were being merged during the trial set-up. The Clinical Trials Unit at University 4 had recently had an MHRA inspection in autumn 2015, and the learning from that recent inspection led to further discussions concerning sponsorship arrangements, delegated duties and the wording of the contract to clarify the role of the NHS sites, which resulted in further delay.

There were delays with the agreement between University 1, University 5 and NHS Site F regarding delineation of the roles and responsibilities of the NHS site and University 5, which included the costing model for the MRI. These discussions were still ongoing when the study was stopped.

The funder also requested oversight of all the subcontracts before they were signed. Delays with the subcontracts led to delays with recruitment and retention of staff at the trial sites.

### Excess treatment costs

In the United Kingdom, the costs of a pharmacological treatment in an RCT are met by a participating pharmaceutical company or by the participating NHS organisation, and not by the research funder. These ETCs amounted to more than £1000 per participant in the intervention group. In Wales ETCs are managed centrally and were agreed by the Welsh government for the two Welsh sites (A and F), whereas in England individual NHS sites are responsible. An ETC application was submitted to NHS site B in June 2014 and approved on 11th March 2015. In NHS site D an initial application for ETCs was declined owing to insufficient funds. The co-investigators from University 4 led negotiations with both NHS site D and the local Clinical Commissioning Groups (CCGs). Both parties argued that they were not funded to support these ETCs, despite guidance on attributing the costs of health and social care research and development [37]. Following negotiation it was agreed that the costs would be split between the local CCGs and the charitable funds from NHS site D. ETCs were approved for NHS site D on 19th August 2015. Provisional ETCs were agreed for NHS site C, who were told that it would be finalised once research and development approval was given.

### Withdrawal of site

Eight months after initiation, NHS site C reviewed the risk assessment of the trial. The locality has a high incidence of TB, and there had recently been several difficult and complex cases treated locally, which had drawn the attention of the local press and community pressure groups [38, 39]. Adalimumab is known to reactivate latent TB [19], and all patients should be evaluated for TB before commencing treatment. The principal investigator (PI) was worried about the risk of reactivating TB with the initial 80-mg dose of adalimumab and decided to withdraw from the trial. Consequently University 3 also withdrew from the trial. This withdrawal led to a risk review for the other sites, who concluded that available data indicated an acceptable risk of infection in their populations, which was consistent with advice provided in the patient information sheet.

### Research physiotherapist recruitment

Delays in signing subcontracts and setting up sites led to delays in recruiting the research physiotherapists at study sites. In NHS site A a physiotherapist was seconded from the NHS physiotherapy department but was required to return to clinical duties because of staffing shortages. This led to delays in recruiting participants into the trial, and to the loss of potential participants. The availability of research nurses and consultant rheumatologists was limited owing to other clinical commitments, so co-ordination of biological agent counselling and investigations was difficult within the time available.

### Trial recruitment

**Fig. 3:**
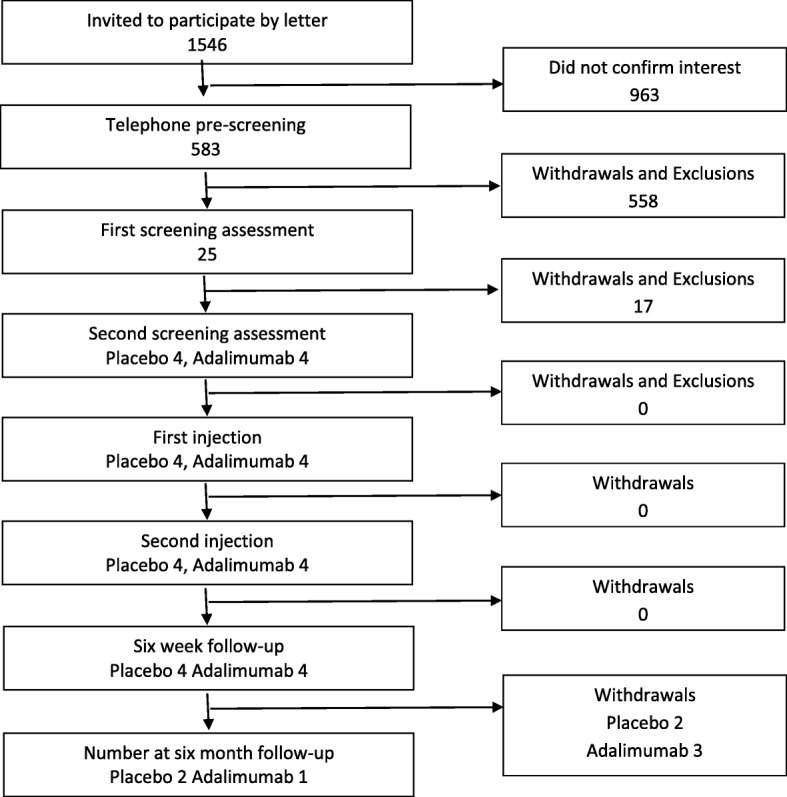
Participant flow diagram for NHS sites A, B, D and E

Recruitment data for the trial are presented in Fig. 3, and reasons for withdrawal or exclusion are listed in Table 2. NHS sites A and B recruited from December 2015 to September 2016. NHS sites D and E recruited from August to September 2016.

**Table 2:** Reasons for withdrawal and exclusion for patients in NHS sites A, B, D and E

Reasons for withdrawal and exclusion from invitation to first clinical assessment	1520
Did not confirm interest	963
No sciatica	210
Symptoms persisting for longer than 6 months	173
Widespread pain throughout body	25
No response or no longer interested	23
No leg pain	20
Complicated symptoms	18
Previous lumbar spinal surgery	16
Trial closed early to recruitment	14
Previous surgery	11
Symptoms resolved/improved	10
Pain in both legs	7
Contraindications to MRI	6
Expressed interest but delay in telescreening due to site staffing issues means no longer meet criteria for inclusion (e.g. no longer in pain or have recently breached the > 22-week exclusion window since replying)	6
Serious spinal pathology	4
Unable to communicate in English or Welsh	3
Mental health problems	3
Current leg pain worse than or as bad as back pain	3
Previous episode of sciatica in the last 6 months	2
Incidental serious pathology identified by MRI	1
Previous use of biological agents targeting TNF-α	1
Contraindications to adalimumab	1
Pregnant or breastfeeding	1
Reasons for withdrawal and exclusion from first clinical assessment to second clinical assessment	17
Mild symptoms – discharged to GP care	7
Study closure	5
Over time limit for second clinical assessment	1
TB screening failed	1
Participant revealed long term history of widespread pain at screening – particularly in shoulders	1
No positive neurological test	1
Patient did not attend appointment and could not be contacted	1
Reasons for withdrawal and exclusion from 6-week follow-up to 6-month follow-up	4
Study closure	4

*Abbreviations: GP* General practitioner, *MRI* Magnetic resonance imaging, *NHS* National Health Service, *TB* Tuberculosis, *TNF* Tumour necrosis factor

Recruitment was less than anticipated. Invitation letters were sent to 1546 potential participants across sites A and B; 583 (38%) were interested in participating and underwent pre-screening by telephone. At pre-screening 210 (36%) did not have sciatica according to our criteria, and 173 (30%) had had symptoms for longer than 6 months, making them ineligible for the trial. Twenty five (4.3%) attended for screening at the first clinic assessment, 13 (52%) were eligible after the second clinical assessment, and 8 were randomised. The other five were eligible but could not be randomised, owing to study closure.

In NHS site A, 16 GP practices identified eligible patients presenting to the practice using database searches or opportunistic referral. Musculoskeletal clinics and physiotherapy departments also searched for eligible patients presenting to their clinics.

NHS site B recruited mainly from a secondary care back pain service rather than from primary care and had a higher rate of exclusion because of longer duration of symptoms (19% of those excluded) than in the other sites (NHS site A, 4%; NHS sites D and E, 5%). It was noted by the PI at this site that referrals of patients with sciatica to the clinics had decreased between planning stages and the start of trial recruitment, owing to a change in the referral pathway commissioned by the local CCG. Therefore, the planned recruitment pathway was changed to inviting GP practices to identify eligible patients by database search or opportunistic referral, independent of referral to specialist services. Database searches commenced at 12 practices in June 2016; 756 potential participants were identified by GP practices, 11 were invited to first clinical assessment screening, and 5 provided initial consent to participate before the trial was terminated.

NHS sites D and E had opened to recruitment on 11th August 2016, but the trial was closed on 26th September 2016. During this period there was a reasonable response rate of 14 of 43 invitations to participate, and nearly half of these, 6 of 14, were made by GPs handing out trial information packs during primary care consultations. This method of recruitment could have been more successful, but we were unable to test this properly before the trial closed. One patient who was eligible after assessment by the research physiotherapist was not able to participate because of trial closure.

### Adverse events

No adverse events or adverse reactions were recorded for any of the eight participants.

## Discussion

### Summary of lessons learnt

This study has several important findings with major implications for developing evidence, within a UK setting, for challenging, expensive interventions with the potential for rare but significant side effects. We found that treatment pathways for acute sciatica varied across research sites and changed during the study period. This necessitated a flexible and heterogeneous approach to study recruitment, matching local treatment pathways. It was possible to introduce a novel treatment approach (biologic therapy) requiring specialist services (rheumatology), not normally a part of existing treatment pathways, within the context of a clinical trial. However, delivering this RCT was challenging, involving multiple professional groups across different health care organisations. In the future, additional feasibility studies, more efficient site set-up, improved and pilot-tested recruitment methods and longer recruitment periods might be appropriate.

There were four main factors that led to delays and early trial closure: contracting issues, securing the payment of ETCs, site withdrawal due to concerns about reactivating TB in a highly prevalent area, and a complex trial recruitment process that did not always match local care pathways. There were long delays agreeing and exchanging subcontracts with participating centres and sites, and contractual discussions with one site were never concluded. Earlier agreement between sponsor, university centres and NHS sites might have been facilitated by the use of model agreements such as the Brunswick research collaboration agreement [40]; site feasibility questionnaires; or research infrastructure that could facilitate the contracting process in multi-site research, such as the National Institute of Health Research Translational Research Partnership [41].

Negotiations for the ETCs were protracted in England, where responsibility for these costs had to be negotiated with different NHS organisations with competing cost pressures; new arrangements are needed [42]. One site withdrew from the RCT before starting recruitment because of a change in the PI’s perception of acceptable risk in the local population, fuelled by recent high-profile media cases. Further discussions between the trial management group and the local PI around potential risks, related concerns and the degree of equipoise might have prevented site withdrawal.

During trial set-up new national management guidance was published [6], as well as a new national back pain and radicular pain pathway [7]. In one site the local sciatica management pathway changed around the time that it opened to recruitment. This site initially relied only on referrals to its secondary care musculoskeletal service but later involved the primary care research network, which was starting to identify participants just before trial closure. In the other open site there were operational issues with identifying the research physiotherapist resource and fitting the trial around the clinical commitments of participating clinicians.

The main method for identifying participants was retrospective GP record review, but this had a low rate of response, with only a small proportion seen at the screening assessment. We had modelled the numbers of eligible participants for our recruitment projections on the previous Assessment and Treatment of Leg pain Associated with the Spine (ATLAS) cohort study, which identified patients in real time as they were consulting, with retrospective record review used only as a backup [43]. Although we identified large numbers of potential participants, only small numbers returned reply slips indicating a willingness to participate. It is not known why potentially eligible participants did not wish to participate. Informal feedback suggested that some patients might have been much improved by the time they were contacted about the trial; some might have found the trial procedures too burdensome, such as the complex two-stage recruitment process; whilst others might not have wished to participate in an RCT, especially in a clinical trial of an investigational medicinal product involving a medication with significant potential adverse effects. Greater patient and public involvement could offer insights into how to explore this. Two of the clinical sites were going to recruit participants using the same methods as the ATLAS cohort, which have been used successfully in another RCT of primary care-delivered treatment for sciatica [44]. However, these two sites signed their contracts just prior to trial closure. Although potential participants had started to be identified, there was insufficient time to recruit them.

The current management of RCTs within the United Kingdom has emphasised recruitment efficiency and delivery of outcomes within short timelines [45]. This remains appropriate for treatments that fit within existing treatment pathways; when they do not, a new pathway must be developed specifically for the trial. In the current study we introduced medical screening and biologic therapy administration delivered through experienced secondary care rheumatology services. The heterogeneity of existing clinical pathways for sciatica (in primary and in secondary care) necessitated a multifaceted approach, with different solutions for different sites, requiring flexibility when pathways at single sites changed between the planning and execution of the trial.

### Comparison with previous literature

The previous systematic review of biological agents for sciatica found a small number of RCTs and other studies with small numbers recruited [17]. Many of these studies also had poor rates of recruitment, both in the UK NHS [46] and elsewhere in Europe [47].

Slow or inadequate recruitment to publicly funded multicentre RCTs is still a common problem [48]. Systematic reviews of RCTs that compared methods to increase trial recruitment found that effective interventions included telephone or text reminders 2 weeks after receiving the letter of invitation, the use of lay advocates who were already involved in the study, monetary incentives, and non-blinding of trial participants. The evaluation of recruitment strategies within RCTs was advocated [49, 50]. Results of a systematic review concerning the recruitment activity of clinicians in RCTs include the use of qualitative research to identify and overcome recruitment barriers, reduction of clinical workload associated with participation in RCTs, extra training and protected research time [51].

### Implications for future research

We make a number of recommendations for future researchers (Table 3). A number of these are pertinent to all RCTs conducted in the United Kingdom. For example, we would recommend full discussions between the sponsor’s contracting department and all university centres and NHS sites to obtain early agreement about what the contracts need to include and how the contracting process should be arranged, so that the university centres and the NHS sites have a clear understanding of their delegated roles and tasks. This may involve model contracts such as the Brunswick research collaboration agreements, which have been designed to be suitable for the majority of cases where two or more universities receive a joint research grant [40]. Early discussions about site requirements, perhaps using a site feasibility questionnaire, early dialogue with sites’ research and development departments, and the early appointment of research staff in each site would facilitate trial set-up.

**Table 3:** Ten lessons learnt for consideration in a future trial

Contracts	1	Early agreement between sponsor, NHS sites and university centres about how the contracting process should be arranged with model research collaboration agreements
Site set-up	2	Early discussions about site requirements using a site feasibility questionnaire
	3	Recruitment of a dedicated research physiotherapist (or other personnel) at each site
Treatment acceptability	4	Establish if the proposed treatment is acceptable to all principal investigators
	5	Determine if the proposed treatment is acceptable to sciatica patients, using further qualitative research
Recruitment	6	Simplify two-stage recruitment process
	7	Use telephone or text reminders two weeks after patients receive letter of invitation
	8	Use of lay advocates already recruited into the study
	9	Recruitment during real-time GP consultations
Feasibility study	10	Feasibility study testing several key recruitment methods

*GP* General practitioner, *NHS* National Health Service

We also make recommendations pertinent to the circumstances of this particular RCT. The impact of research staff shortages, in this case a research physiotherapist at one site, could be avoided by having dedicated research staff. In addition, involvement of the research staff during the initial planning stage would have been useful for planning the recruitment strategy.

Further qualitative research is needed to identify reasons for low recruitment rates, using methods such as the QuinteT Recruitment Intervention, which uses a combination of standard and innovative qualitative research methods, with some simple quantification, to understand recruitment and identify sources of difficulty [52]. Possible reasons for poor recruitment include concerns about the nature of the trial intervention and its side effects, perceived burden of trial participation, natural history of recovery of severe sciatica, perceptions about the nature of sciatica itself, and whether the treatment under study is consistent with these. People who believe that their sciatica will resolve quickly (either spontaneously or with treatment) are unlikely to commit to a trial of medical intervention with long follow-up, particularly if they perceive that it would not provide (and might delay) definitive treatment. Such beliefs in the study population might not be well-founded in evidence, and pre-recruitment education might be necessary to help potential participants appreciate the possible benefits that might be achieved from novel interventions that are being investigated.

Patient recruitment from ‘real-time’ GP consultations may have reduced the delays associated with retrospective checks of GP consultations and from referrals to physiotherapy and secondary care settings. Unfortunately, because of delays in agreeing ETCs and finalising contracts, there was insufficient time to recruit any participants using this method before trial closure.

## Conclusions

A trial of biological therapy in patients with sciatica still needs to be performed, but it would require a clearer contracting process, qualitative research to ensure that patients (and clinicians) would be willing to participate, and more efficient recruitment methods, with the least possible burden on patients.

## Abbreviations

ATLAS: Assessment and Treatment of Leg pain Associated with the Spine
CCG: Clinical Commissioning Group
EQ-5D: European Quality of Life-5 Dimensions 5-level version
ETC: Excess treatment cost
GP: General practitioner
HTA: Health Technology Assessment
MHRA: Medicine and Healthcare products Regulatory Agency
MRI: Magnetic resonance imaging
NHS: National Health Service
ODI: Oswestry Disability Index
PI: Principal investigator
R&D: Research and development
RCT: Randomised controlled trial
REC: Research ethics committee
TB: Tuberculosis
TNF-α: Tumour necrosis factor-α
